# Accelerated high-throughput imaging and phenotyping system for small organisms

**DOI:** 10.1371/journal.pone.0287739

**Published:** 2023-07-21

**Authors:** Talha Kose, Tiago F. Lins, Jessie Wang, Anna M. O’Brien, David Sinton, Megan E. Frederickson

**Affiliations:** 1 Department of Mechanical and Industrial Engineering, University of Toronto, Toronto, Ontario, Canada; 2 Department of Ecology and Evolutionary Biology, University of Toronto, Toronto, ON, Canada; 3 Department of Molecular, Cellular, and Biomedical Sciences, University of New Hampshire, Durham, NH, United States of America; King Abdulaziz University, SAUDI ARABIA

## Abstract

Studying the complex web of interactions in biological communities requires large multifactorial experiments with sufficient statistical power. Automation tools reduce the time and labor associated with setup, data collection, and analysis in experiments that untangle these webs. We developed tools for high-throughput experimentation (HTE) in duckweeds, small aquatic plants that are amenable to autonomous experimental preparation and image-based phenotyping. We showcase the abilities of our HTE system in a study with 6,000 experimental units grown across 2,000 treatments. These automated tools facilitated the collection and analysis of time-resolved growth data, which revealed finer dynamics of plant-microbe interactions across environmental gradients. Altogether, our HTE system can run experiments with up to 11,520 experimental units and can be adapted for other small organisms.

## Introduction

Scientific dataset size has increased over time, as larger datasets allow detection of more or more-complex effects. However, data demand has skyrocketed with the rise of artificial intelligence (AI) and machine learning (ML). AI and ML have transformed inference but depend on expansive training datasets. High-throughput experimentation (HTE) is key to creating large databases for AI and ML [1]. The primary challenge in HTE is the substantial investment in adapting existing experimentation methods to novel automated systems, which can dissuade researchers. Yet, pre-automation methods generally depend on intensive manual labor, creating bottlenecks and higher costs over the long term. Therefore, functional and cost-friendly HTE systems that are compatible with existing experimental methods and adaptable across experimental systems can substantially advance scientific research [2].

Biological research often seeks to understand multiplicative effects of organism types (e.g., multiple species or genotypes) across environments. Yet, the required experiment scale exponentially increases when considering interactive effects of multiple organism types [3], multiple environments [4, 5], or both [6]. HTE tools that facilitate experiment setup can provide transformative results [2]. Like setup, collecting data from each experimental unit is frequently rate-limiting [7]. HTE data collection in biology often extracts phenotypes via automated imaging and automated image processing to increase speed [8, 9]. Error detection and elimination during HTE image processing maintains accuracy [10] and is equally critical to overcoming phenotyping bottlenecks. Feature extraction algorithms built with computing languages can even extract specific phenotypes, such as leveraging color thresholding to detect plant tissue characteristics [11, 12].

Research on host-microbiome interactions has a growing issue of experimental scale [2]. Like most species interactions, host-microbiome interactions often vary across environments [13, 14]. Yet, environments vary in myriad ways, presenting a real challenge to biologists. Scientists often want to measure host phenotypes, host fitness, or turnover in microbiome composition while manipulating several environmental variables together and individually, but rate-limiting steps restrict designs [15].

Researchers must consider both growth rate and utility to environmental applications when selecting a host-microbiome study system. Duckweeds hold promise as a model system due to existing genomic resources, their rapid growth across many conditions, and their diverse applications including bioremediation, nutrient recovery from wastewater, biofuel, and nutrition for animals or even humans [16–20]. Duckweeds, including *Lemna minor*, are aquatic with tiny floating fronds. As mother fronds produce daughter fronds, the generations can remain attached, resulting in clusters ~1–15 mm in diameter [21]. Handling small floating duckweeds on liquid surfaces at the HTE scale can overwhelm even experienced researchers, creating a major bottleneck. One new approach uses filtration systems, and another uses a special coating on inoculation loops to pick and move duckweeds but requires an unrealistic solid media [22]. Unfortunately, both solutions are insufficient for duckweed HTE due to incompatibility with existing labware.

Duckweeds are potentially practical in HTEs with automated image-phenotyping due to their tiny size. However, duckweed experiments using images remain small-scale. Caicedo et al. [16] reports only 10 treatments with three replicates. One barrier is consistency. Only replicable imaging conditions yield the necessary standardization for batch processing (8), but these have not been realized at the HTE scale for duckweeds. Even without imaging or phenotyping, the highest capacity duckweed experiment currently reported is 3,000 units [23]. Automated imaging systems where duckweeds can be maintained inside a standardized platform should provide the path forward.

We developed automated systems that accelerate experimental setup and phenotyping, allowing truly high-throughput duckweed experiments. Our customized duckweed loading system integrates a liquid-handling robot to ease labor-intensive setup. Our automated imaging system takes images (or videos) of samples throughout the experiment without interruption, while our user interface runs color-thresholding in fast, labor-free image-phenotyping. When combined, these tools enable experiments with up to 11,520 duckweed units. To demonstrate our system, we ran an experiment with 6,000 duckweed units. Although we focus on duckweeds here, our system can be adapted to various small organisms.

## Materials and methods

### Experimental design

We investigated the effects of nutrient stressors on duckweed growth over time. Ten concentrations each of NaNO_3_, Ca(H_2_PO_4_)_2_, and KCl were crossed to generate 1,000 combinations of nitrogen (N), phosphorus (P), and potassium (K) levels. These combinations were crossed with two microbe treatments: present (inoculation with microbes isolated from the plant line) or absent (inoculation with sterile media). We replicated these treatments using one genotype of the duckweed *Lemna minor* to reach 6,000 units.

### Autonomous duckweed loading system

The biggest challenge of a HTE duckweed experiment is loading duckweeds into multi-well plates. It is tedious to pick up duckweed fronds from a source jar since they are floating freely on the liquid surface and small in size. An experienced researcher can fill a 96-well plate with duckweeds and other experimental components in 2–3 hours, but the job is highly repetitive. To reduce the manual labor involved, we modified an Opentrons-2 (OT-2) liquid handling system to load wells with duckweeds. This system could already fill well plates with liquids in custom experimental designs, was already integrated with a HEPA filter to sustain a sterile environment, and has a gantry system appropriate for handling duckweeds. Protocols can be customized using the manufacturer’s web-based tool or Python API.

When working manually, disposable plastic inoculation loops can be used to pick up duckweeds from a source jar, but there are no commercially available loops for the OT-2. We modified commercial inoculation loops (BioPlas, 1 μL Astral inoculation loop, 7000) with 10 μL pipette tips to work with OT-2 P300 pipette heads (details in S1 File). Capillary action between the loop and the liquid surface causes the duckweed to climb on the loop and stick to it. The destination wells must be pre-loaded with liquid for the release of the duckweed from inoculation loops moved by the OT-2.

**Fig 1:**
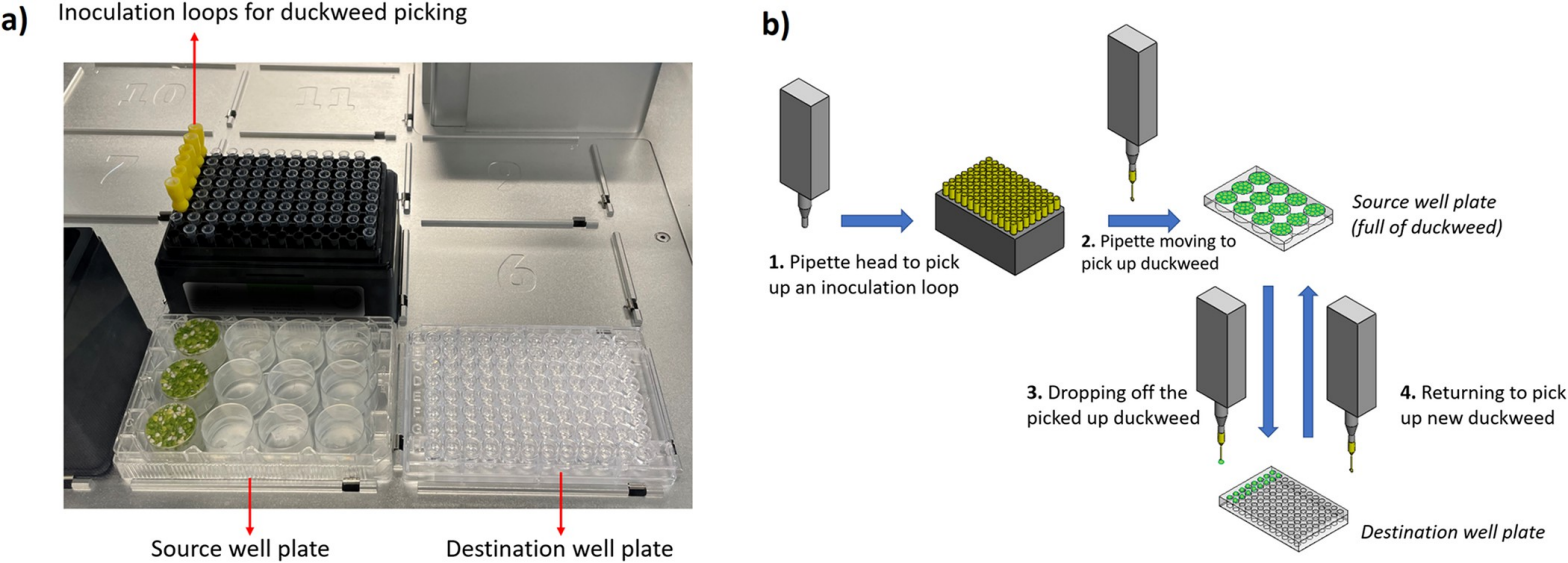
Duckweed loading system. Layout of the autonomous duckweed loading system presented in a), and schematic of the protocol sequence demonstrating the duckweed loading operation presented in b).

Using the OT-2, we filled 96-well plates with duckweeds by transferring them from a 12-well ‘source’ plate to destination wells. The entire experiment can be set up with the OT-2 (Fig 1), accelerating the set-up stage; details (S1-S3 Figs in S1 File) and a video showing duckweed loading are in the S1 File. High density of the duckweeds and precise level of DI water in the source wells were critical for effective transfer.

### Autonomous imaging system

**Fig 2:**
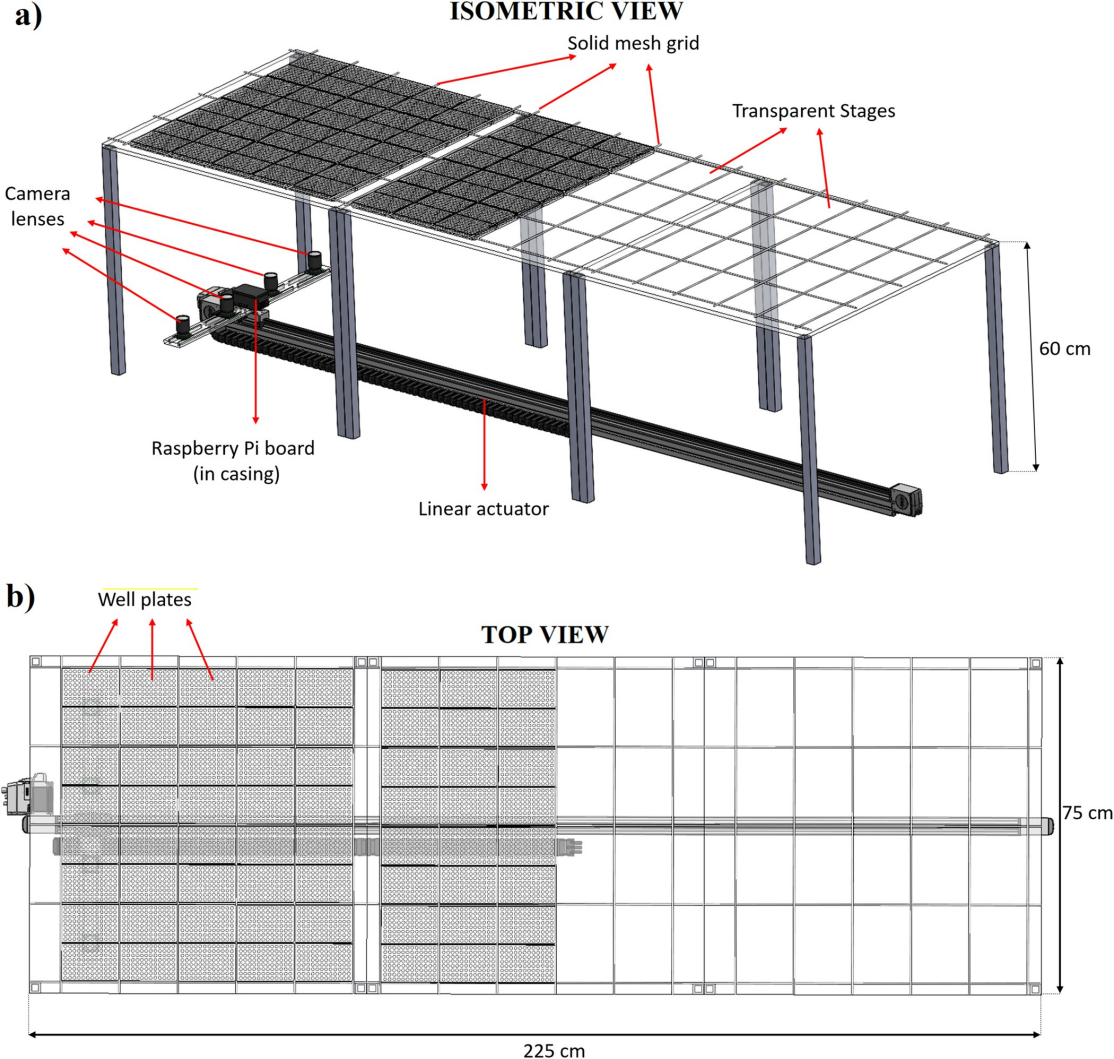
Autonomous imaging system built in a growth chamber in isometric view a), and in top view b). The cameras take images from below the transparent stages holding plates. The grid on the transparent stages prevents plate misalignment. The linear actuator carries the imaging system to complete the imaging.

The imaging system consists of four high resolution (12.3 MP) cameras (Raspberry Pi High Quality Cameras) multiplexed over a Raspberry Pi 4 board, a camera holder, a linear actuator (Zaber Technologies, LC40B2000), and three transparent stages that hold the experimental well plates full of duckweeds. The system sits inside a growth chamber, functioning as a photo booth (see Fig 2, S4, S5 Figs in S1 File).

The cameras and the Raspberry Pi board were bolted on the linear actuator via the camera holder, while the 96-well plates were placed on the transparent stage. The position of the camera lenses is adjustable using the camera holder slots. The transparent stages are 95% transparent acrylic, 75 cm x 75 cm in footprint, and 1 cm thick. A solid mesh grid, which was made of acetal rods and built on the transparent stages using epoxy, was utilized to fix the position of well plates. The imaging system was located under the transparent stages, enabling image capture from below the well plates.

When activated, the four cameras sequentially photograph the well plates in one row, then the linear actuator moves them to the next. Each image captures two well plates (4056 x 3040 pixels per image, 175 x 175 per well), allowing 8 well plates per row and enough image detail to measure duckweed phenotypes. The linear actuator and cameras capture photos of all plates in under 10 minutes. Considering growth camber size, actuator travel range (2 m), and standard 96-well plate dimensions, the imaging system can monitor 120 well plates, or up to 11,520 experimental microcosms.

### Autonomous phenotyping tool

**Fig 3:**
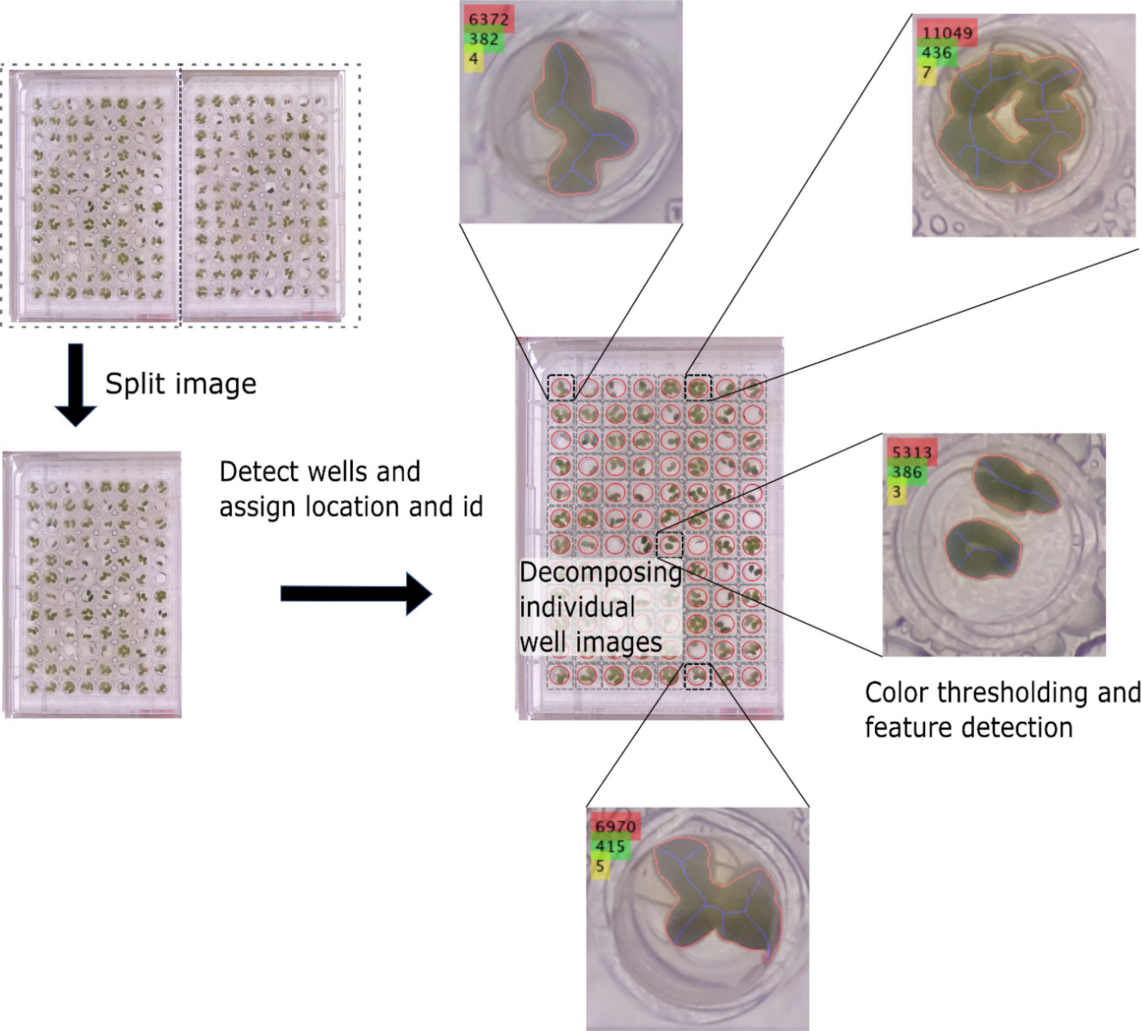
Workflow of the phenotyping tool. First, the raw image containing two well-plate images is split into individual well-plate images. Then, the wells are identified, and decomposed into individual images. Finally, color thresholding extracts duckweed features in a well including frond area, number, and greenness.

Our image processing tool leverages MATLAB graphical user interfaces (GUIs, S6, S7 Figs in S1 File). First, we extract single 96-well plate pictures from raw images. Plate images are then decomposed into images of each well by anchoring a binary mask grid of 96 circles (one per well) with a Hough transform that locates well outlines in the plate image. We next combined HSV-based and green intensity color-thresholding for each pixel to extract frond area and number of duckweeds per well (Fig 3). Our code also examined duckweed “greenness,” which distinguishes unhealthy (yellow or pale) from healthy (bright green) fronds and is associated with leaf nitrogen content in other plants [24, 25]. Calculation details and supplementary systems to handle post-processing hurdles are in the S1 File.

## Results

Our automated duckweed loading system prepared an experiment with 6,000 microcosms of duckweeds in 2,000 nutrient and microbe treatments in 64 96-well plates. First, an OT-2 system loaded experimental media using the system’s standard tools. Then, the OT-2 system outfitted with our new duckweed-picking tool, filled the plates with duckweeds. In duckweed loading, up to 87.5% of wells were filled with at least one duckweed, with an average fill rate of 75% (S3 Fig in S1 File). The whole protocol to fill one 96-well plate takes 13 minutes. The 13–30% empty wells were manually filled by the researcher prior to sealing plates with gas-permeable membranes.

The autonomous imaging system photographed all units every day at 9 pm for 10 days and stored the photos in its memory. The raw files were processed by the autonomous phenotyping tool. Further data analysis and visualization was performed in R version 4.2.1 (R Core Team, 2022). Model descriptions and results tables are in the S1 File.

**Fig 4:**
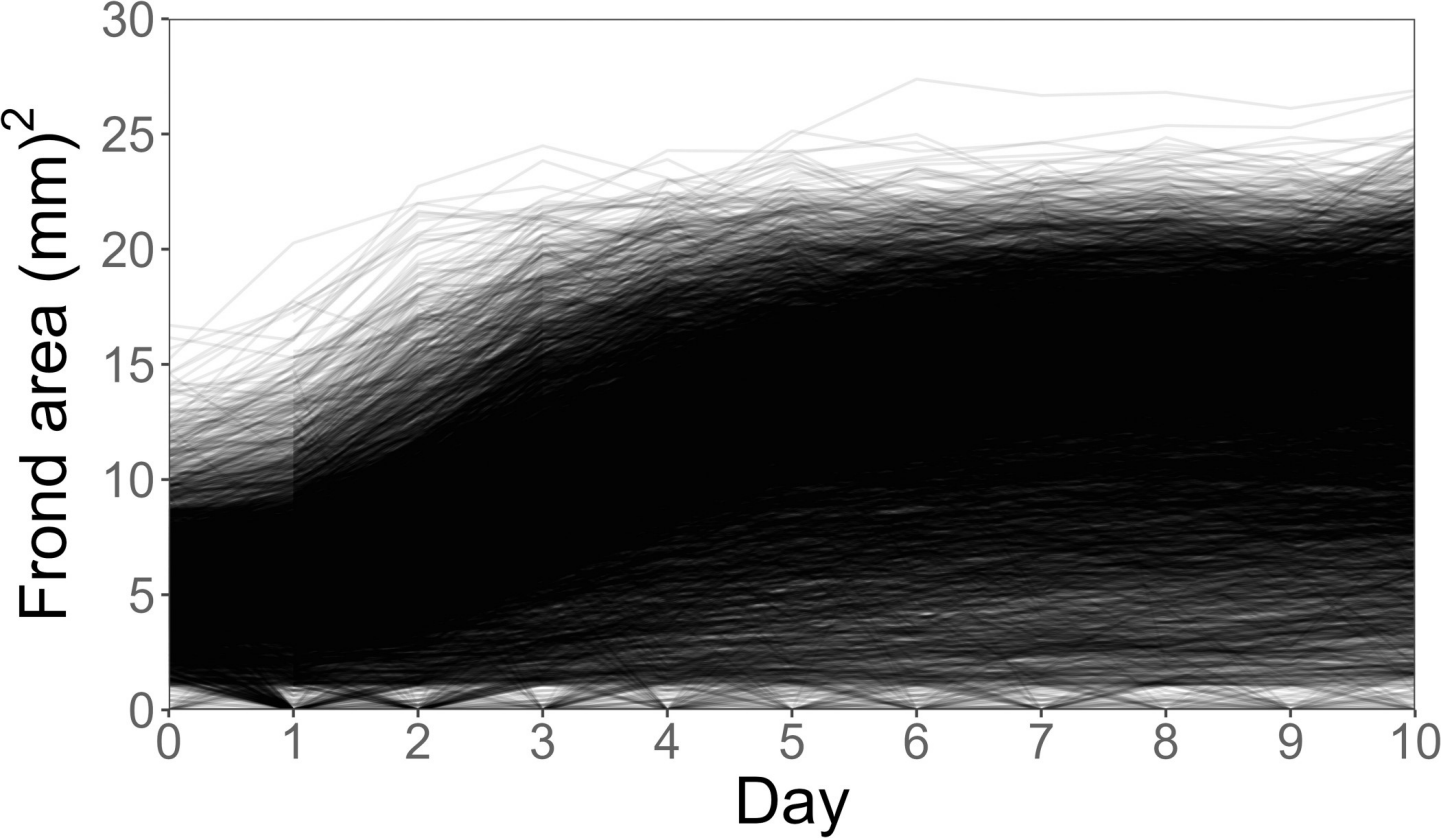
Growth of L. minor in all 6,000 experimental units through time. Frond area in all wells was measured once per day, and each line connects the area measurements of a single well across time. Lines for all 6,000 wells are plotted on top of each other, such that the high-density area (black, many lines plotted on top of each other) depicts the most common growth trend across all experimental units. Individual wells sometimes appear to grow from zero to positive frond area–these must have always contained some living tissue, as no new duckweeds were added. Wells could be scored as having zero living tissue if the living tissue did not meet the size or color thresholds in the processing software. For example, fronds infrequently do recover from initial yellowing, or, if they die, their internally held daughter fronds may remain alive and become visible in one or two days.

The system successfully tracked plant growth through time (Fig 4, S1 Table in S1 File), which would have been challenging and labor-intensive to do manually. Duckweeds grew quickly (large positive effect of Day: p < 0.001). While total frond area continued increasing throughout the experiment, growth started to plateau around day 5 (Fig 4, negative effect of Day^2^: p < 0.001). This may be explained by the onset of frond death, or well boundaries restricting frond growth.

**Fig 5:**
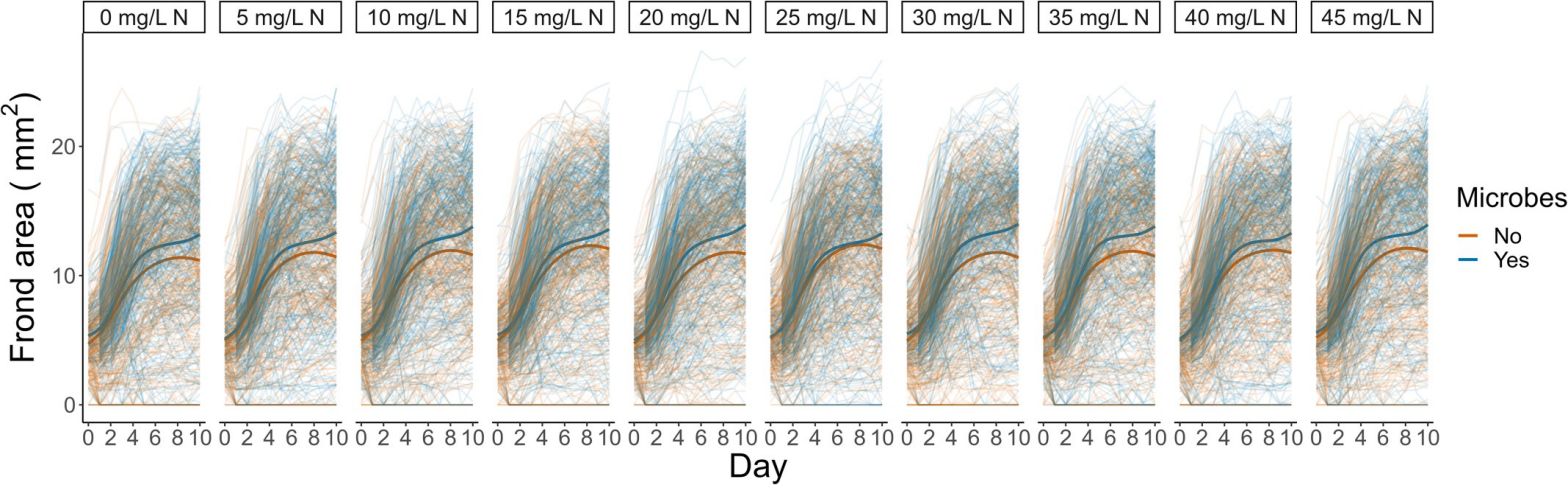
Growth of duckweeds across nitrogen levels. Each line is frond area in one well. Darker lines depict smoothed conditional means of plants inoculated with microbes (blue) and plants not inoculated with microbes (orange), averaged across other nutrient levels.

In the absence of microbes and across levels of other nutrients, more N increased plant growth, except at the highest levels of N, which reduced growth (Fig 5). Plants inoculated with microbes grew faster overall, and microbes shifted the N level that supported the most duckweed growth and prevented the growth decline at higher N (positive interactive effect between microbes and the square of nitrogen level, S1 Table in S1 File). In the absence of microbes, duckweeds grew most at 25 mg/L N. In plants inoculated with microbes, duckweeds grew most at 30 mg/L N (Fig 5). The data collected by the HTE system also revealed time-resolved effects on frond greenness, allowing a finer understanding of non-linearity in duckweed response to varying nitrogen levels and microbes (S8 Fig, S2 Table in S1 File). Complete interactive results of N, P, K, and microbes are reported in [26] (in preparation).

## Discussion

The successful integration of our automated duckweed loading system, autonomous imaging system, and autonomous phenotyping tool enabled a high-throughput duckweed experiment with 6,000 units. Across our two biological response variables, high experimental throughput and automated phenotyping allowed a deeper understanding of the complex interactions between duckweeds and their environment. The time-resolved phenotype and growth data that our platforms provided allowed finer-scale detection of time-resolved effects across a high number of treatments.

Our automated duckweed loading system expedited the preparation process. The current open-loop version of the OT-2 does most of the manual labor, with a success rate as high as 87.5%. The researcher could supplement missed (empty) wells by hand or simply increase replicates of each treatment to allow for some missed wells, leveraging the advantages of the high-throughput system. We decided not to pursue closed-loop capability given the ease of open-loop implementation, marginal improvement possible, and work required. Closed-loop capability could theoretically be realized with image-processing-based feedback that programs the robot to re-attempt failed duckweed transfers.

Our autonomous system images wells automatically without disturbing the experiment but was not developed without challenges. The vibration of the growth chamber’s environment support systems caused slight image distortion, which also slightly impacted frond area calculations by as much as ±4.7% (standard deviation of frond area in five images divided by the mean). To minimize this distortion effect, we took five consecutive images and averaged their post-processing values.

Our autonomous phenotyping tool smoothly extracted data in most cases. However, sometimes duckweed color was near our pre-defined color-thresholding limits. This resulted in “zero” frond area one day, and a large non-zero area later with only a slight shift in frond color. Our optimization GUI tailored color thresholding limits to minimize these errors (S7 Fig in S1 File). This GUI used a training set of 250 well images with individually varied color threshold settings that produced satisfactory duckweed area measures. It then identified settings that minimized error in the training set, achieving ~9% error reduction. Future studies could expand the phenotyping tool by leveraging more color information in images, such as by applying vegetation indices [27].

There are other interesting directions in which to develop high-throughput imaging systems for duckweeds in the future, including 3D microscopic imaging [8], fluorescence imaging [28], and microtomography [22]. Perhaps most obviously, our automatic imaging and phenotyping systems could be extended to other organisms. Larger plants could be incorporated with automated tissue-culture or seed-germination image assays. The automated imaging system can also record video and could monitor mobile specimens. Automatic video monitoring and processing would facilitate experiments with tiny invertebrates, such as *Daphnia*, and *Artemia*, or small insects [29]. Indeed, video tracking phenotypes already exist for many such organisms, and would only need to be integrated with our automatic imaging platform.

## Conclusion

Progress in biology requires decomposing multiplicative effects of environmental and biotic variation. These highly multi-factorial endeavors in turn require increasingly vast datasets, which are costly and time-intensive to generate manually. Going forward, we need continued integration of biological model systems into automated platforms, especially flexible platforms that can be adapted across organisms, like our automated imaging and picking methods. We expect biology to progress most with improvements in automated handling of more organism types and their media, and in automated extraction of more phenotypes from images and video.

## Supporting information

S1 File (PDF)
