# Supplementary material for: Accelerated high-throughput imaging and phenotyping system for small organisms
Source: PLoS One. 2023 Jul 21;18(7):e0287739. doi: 10.1371/journal.pone.0287739 (PMC10361482; doi:10.1371/journal.pone.0287739)
Supplement: S1 File — (PDF) [file pone.0287739.s001.pdf]

Supporting Information

Accelerated high-throughput imaging and phenotyping system for small organisms

Talha Kose[1], Tiago F. Lins[1], Jessie Wang[2], Anna M. O'Brien[1,2,3], David Sinton[1], Megan E. Frederickson*[2]

[1] *Department of Mechanical and Industrial Engineering, University of Toronto, 5 King's College Road, Toronto, Ontario, M5S 3G8, Canada*

[2] *Department of Ecology and Evolutionary Biology, University of Toronto, 25 Willcocks St, Toronto, ON, M5S 3B2, Canada*

[3] *Department of Molecular, Cellular, and Biomedical Sciences, University of New Hampshire, 46 College Rd, Durham, NH, United States*

[*] *Corresponding Author Email: m.frederickson@utoronto.ca*

Preparation of Inoculation loops for Automated Duckweed Loading

The modification includes cutting the top part of universal 10 µL tips (Opentrons 10 µL Tips) and attaching them inside inoculation loop tips (BioPlas, 1 µL Astral inoculation loop, 7000) using epoxy. After this modification, all inoculation loop tips are sterilized by soaking in 70% EtOH for five minutes, followed by exposure under ultraviolet (UV) light for 30 minutes. Fig. S1 illustrates how the inoculation loops are modified with the 10 µL tips.

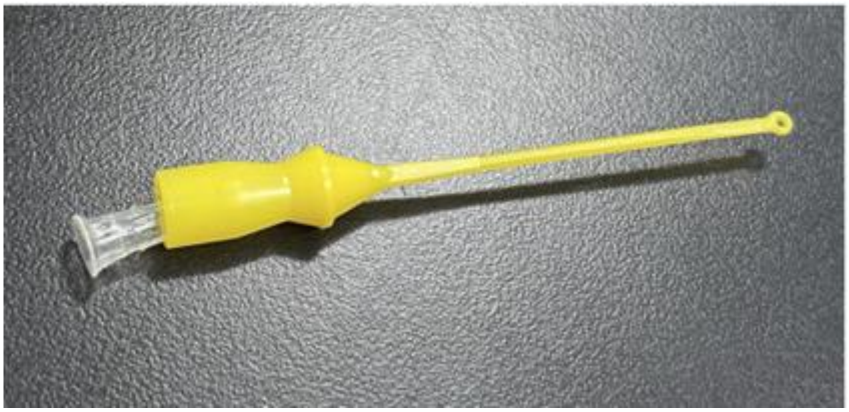

Fig. S1: *Photo of the single inoculation loop and the filter tip that is glued in via epoxy.*

Image of Automated Duckweed Loading System

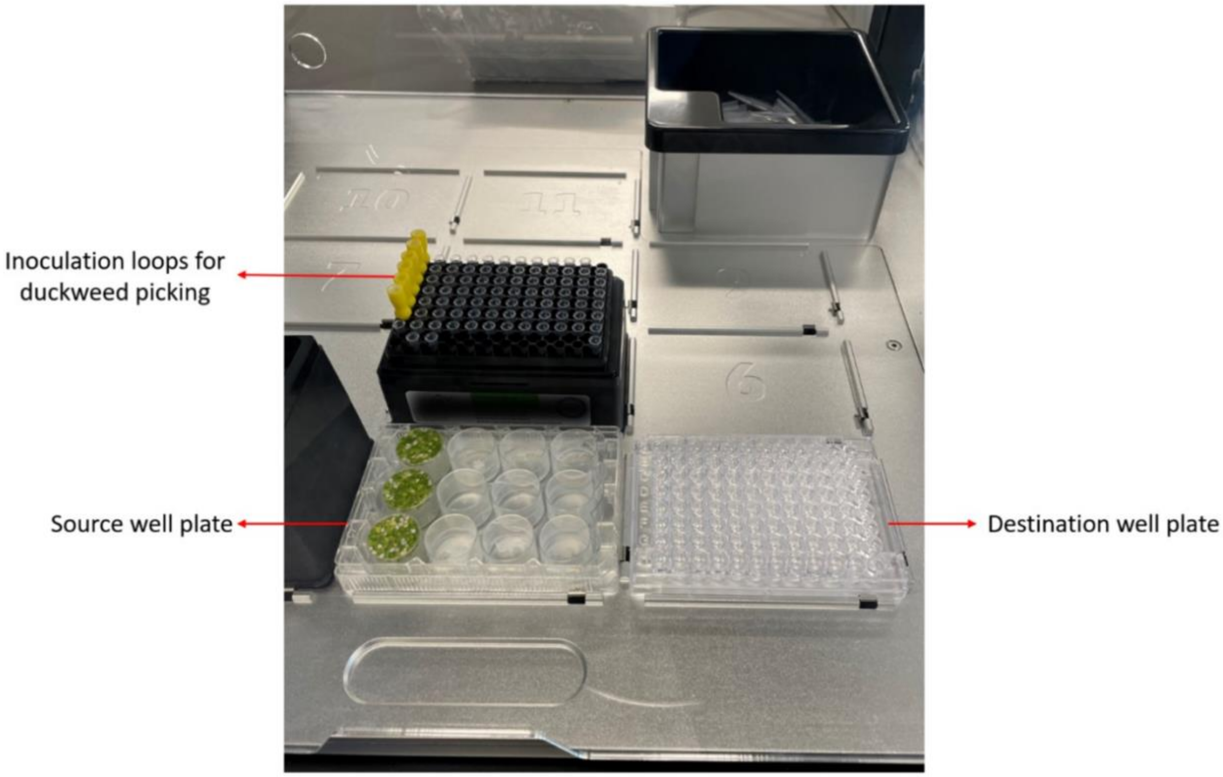

Fig. S2: *Overview of the automated duckweed loading system built in the OT-2 system for single well plate loading. Only one well plate is shown as the destination well plate although the system allows up to nine well plates as destination well plates for further acceleration in the loading process.*

Command Specifications for Duckweed Loading

The protocols to pick up the duckweed from a source well (on a 12-well plate) and drop off to a destination well (on a 96-well plate) were prepared using a web-based protocol designer tool provided by the manufacturer (Opentrons, 2022). Movements already defined in the protocol designer to handle liquids were employed for duckweed loading. We directed the inoculation loop to scan the source well surface with the “Touch tip” command, which passed the loop through where the duckweeds were most crowded in the well, and improved the success of duckweed picking. Dipping the inoculation loop into the destination well was enough to release the duckweed from the inoculation loop. A “Delay” command allowed the duckweed to drift away from the inoculation loop after dipping and before loop removal.

Video of Duckweed Loading

https://doi.org/10.6084/m9.figshare.23549823

Success Rate of Automated Duckweed Loading System

The automated duckweed loading system was tested many times before the actual experiment in order to examine the success rate of duckweed loading. Success was defined as filling the destination well unit with at least one duckweed sample. Fig. S3 shows three destination well plates after consecutive operations with their corresponding success rates.

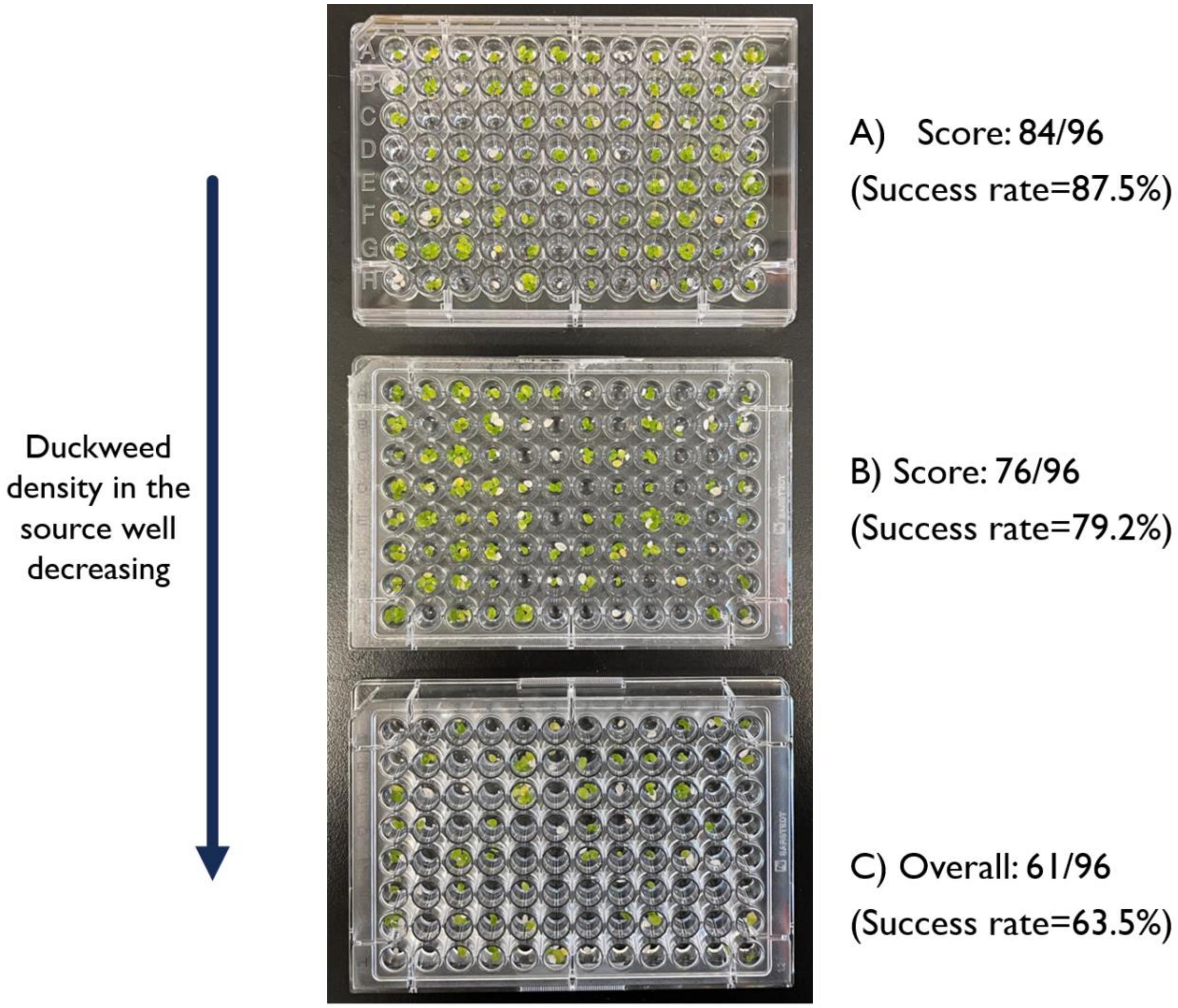

Fig. S3: *Three destination well plates after the automated duckweed loading operation. The success rate drops from 87.5% to 63.5% due to the decrease in duckweed density in the source well as well as the change in the liquid level in the source well. The overall success rate of three well plates was calculated to be 76.7% which is inferred to be the norm of the system.*

The results show that the success rate of our system depends on initial conditions of our source well plate where the duckweeds are packed before the operation. The density of duckweeds in the source well and the liquid level in the source wells are two critical parameters. During operation, the number of duckweeds in the source well reduces due to transfer of the samples. The transfer also affects the liquid level since each duckweed samples also carries some water from the source well. Hence, the researcher using the system should supervise the system

while in operation and potentially supply either more liquid to the system in order to maintain the liquid level or more duckweed after completion of a destination well plate. If properly supplied, the system can deliver a success rate as high 88%. The overall system success is expected to be 75% which is close to the values presented in Figure S3.

Automated Duckweed Imaging System

All photos captured by the cameras are time-stamped and stored in Raspberry Pi memory, which is accessible through remote desktop connection for researchers in the network. The control of the linear actuator and image capturing via Raspberry Pi cameras are automated over a Python script. The whole imaging system can be scheduled to operate autonomously at the desired time using this developed code. Hence, experimental imaging can be uninterrupted and is fully automated.

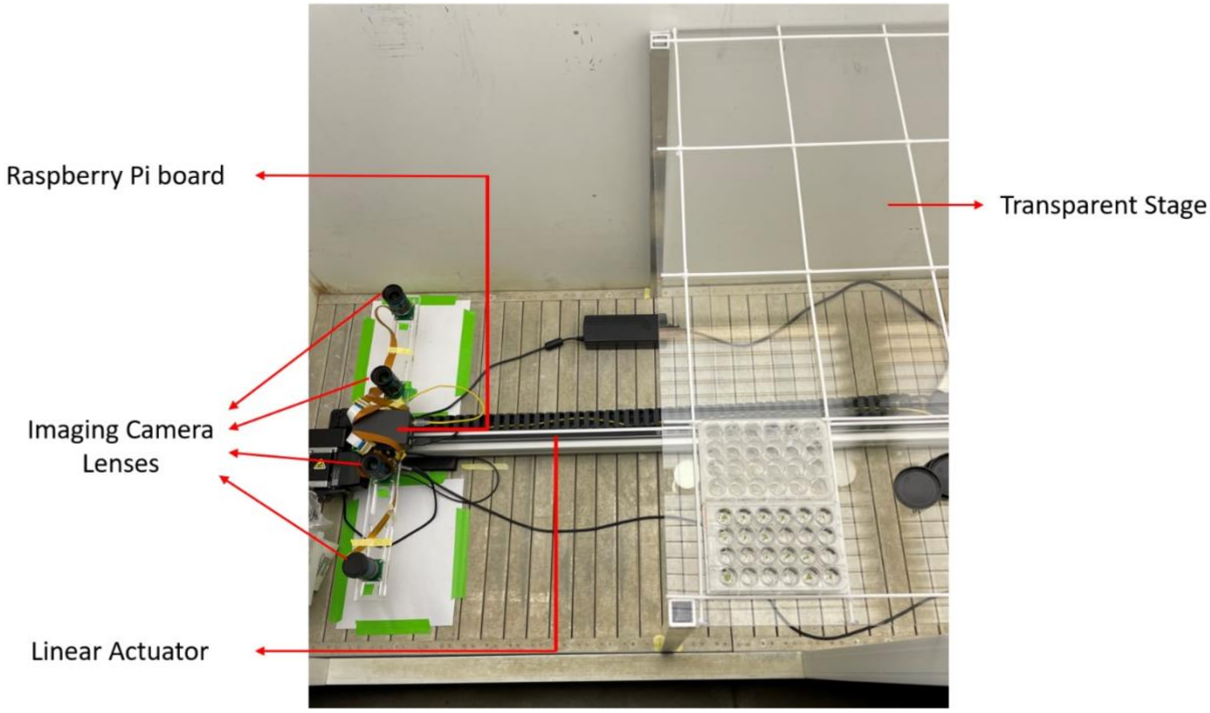

Fig. S4*: Overview of the automated duckweed imaging system built inside the growth chamber. The slots of the transparent stage can host two well plates as seen at the bottom left corner of the stage.*

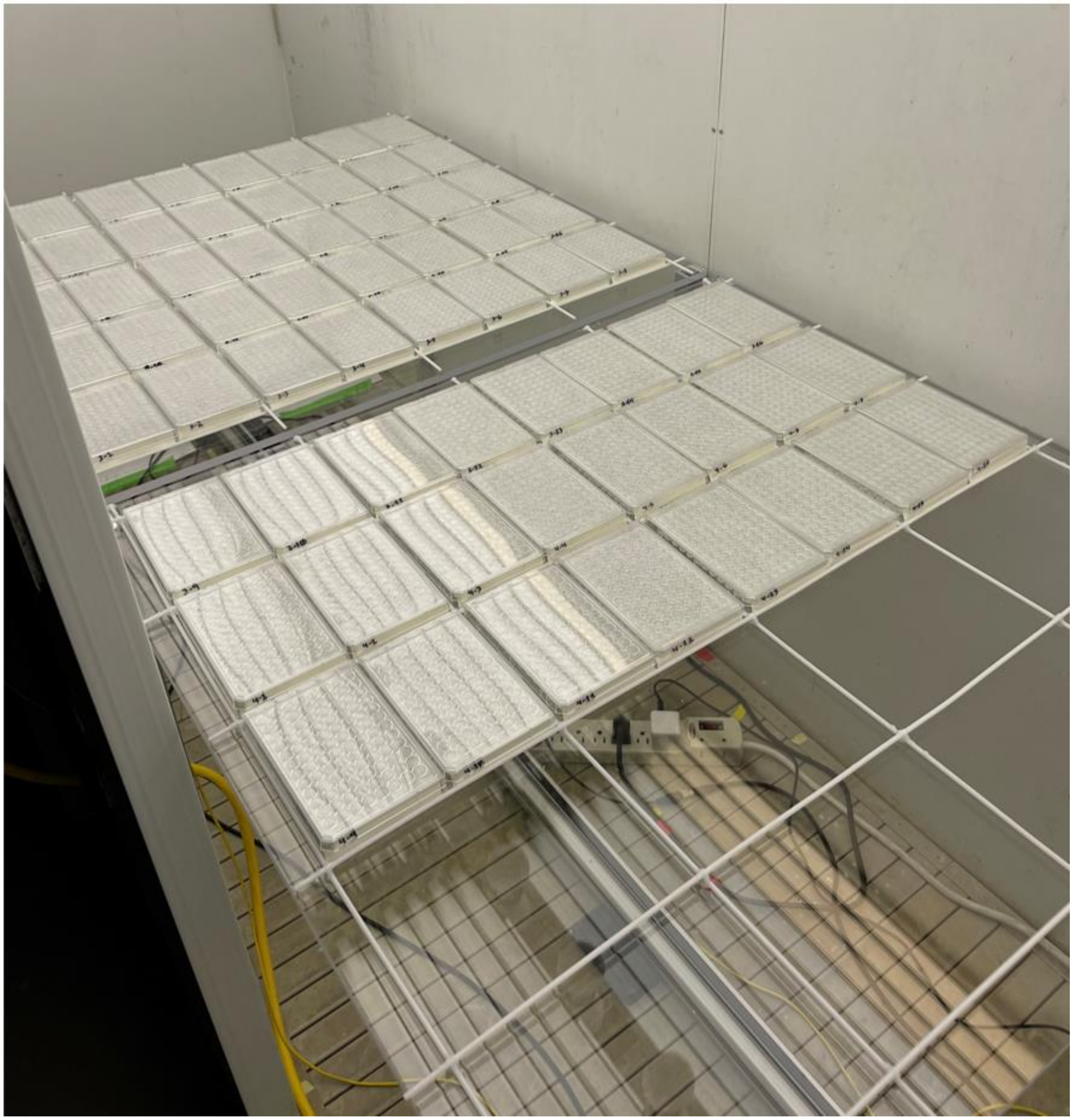

Fig. S5: *The photo of the experimental setup used in automated imaging system. 6,000 duckweed units were used in 64 well plates that can host up to 96 units.*

Supplementary Information on Phenotyping Tools

Phenotypic information gathered from image analysis included area of live fronds (calculated from pixel area using a conversion factor (pixels per well plate length ÷ length of well plate)^2),

frond number, and the greenness of fronds. Green intensity (GI) was calculated for each duckweed pixel as $GI=G/(R+G+B)$, where R, G, B are the red, green, blue values for each pixel, with each ranging from 0 to 255. GI was averaged across pixels in a frond to yield relative frond greenness.

We developed supplementary systems to handle post-processing hurdles resulting from slight deviations in experiment setup, such as poor well image extraction due to imprecise positioning of well plates ("tilted" images), or failing to detect the duckweed in the wells due to tone differences in color (lighting quality variation). The manual well detection interface, again created as a MATLAB GUI, helps researchers adjust automatic well detection results to correct any improper identification of the wells. Similarly, we created another GUI to allow researchers to adjust the automatic color thresholding to minimize the undetected duckweed fronds. This GUI allows the user to create a training sample, then uses a random search algorithm to improve color threshold by minimizing pixel misclassification. Fig. S6 demonstrates the overview of the GUIs. Fig. S7 illustrates the details of the optimization tool workflow.

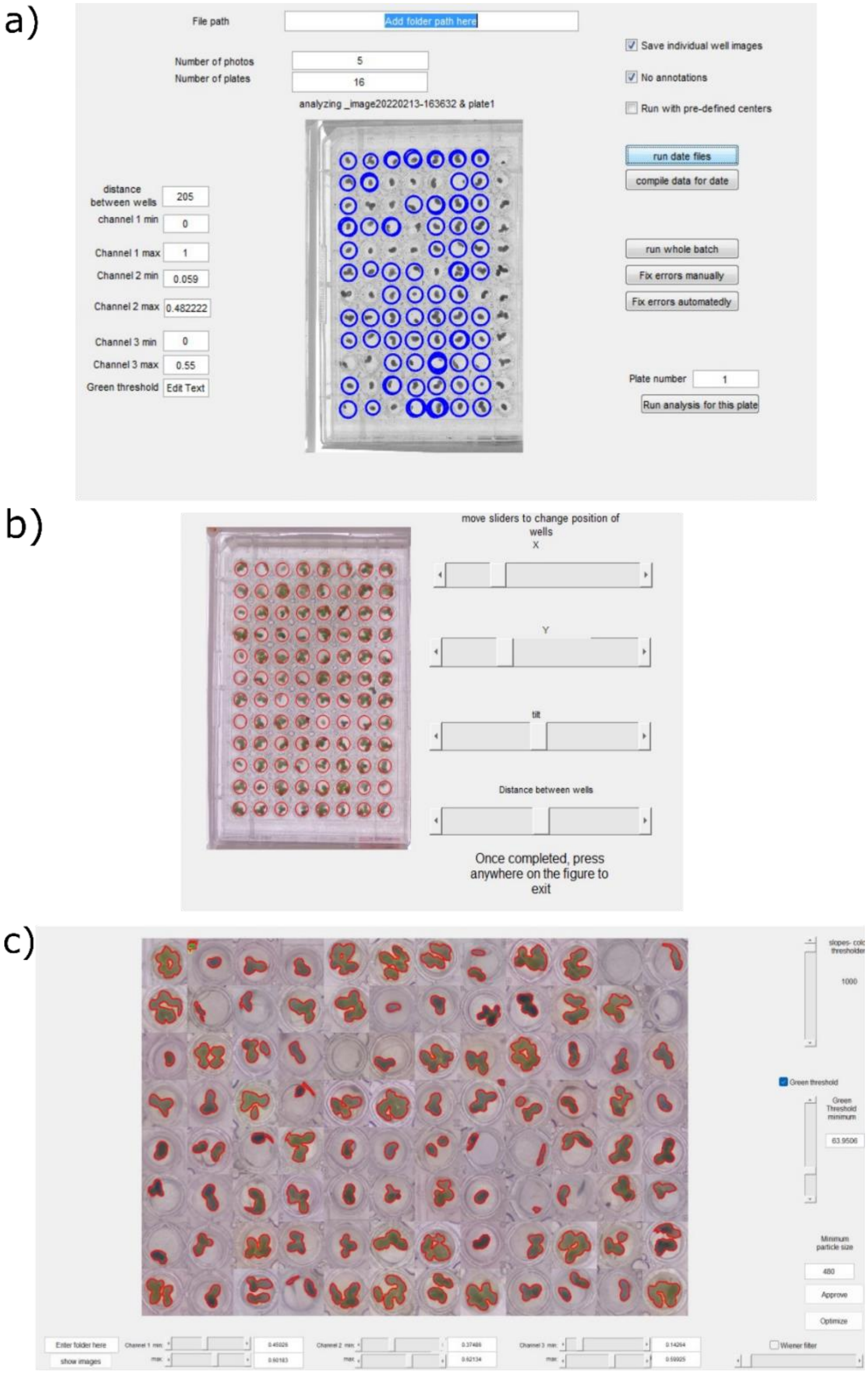

Fig. S6: *Graphical User Interface applications for automated phenotyping: main application to process image files, locate wells in each image and extract features from each well (a); manual correction tool allowing user to manually fine tune the well location to ensure all duckweed area is accounted for in the*

analysis (b); color threshold settings optimization tool offering a visual way to change feature extraction settings and optimize settings using a training sample of images (c).

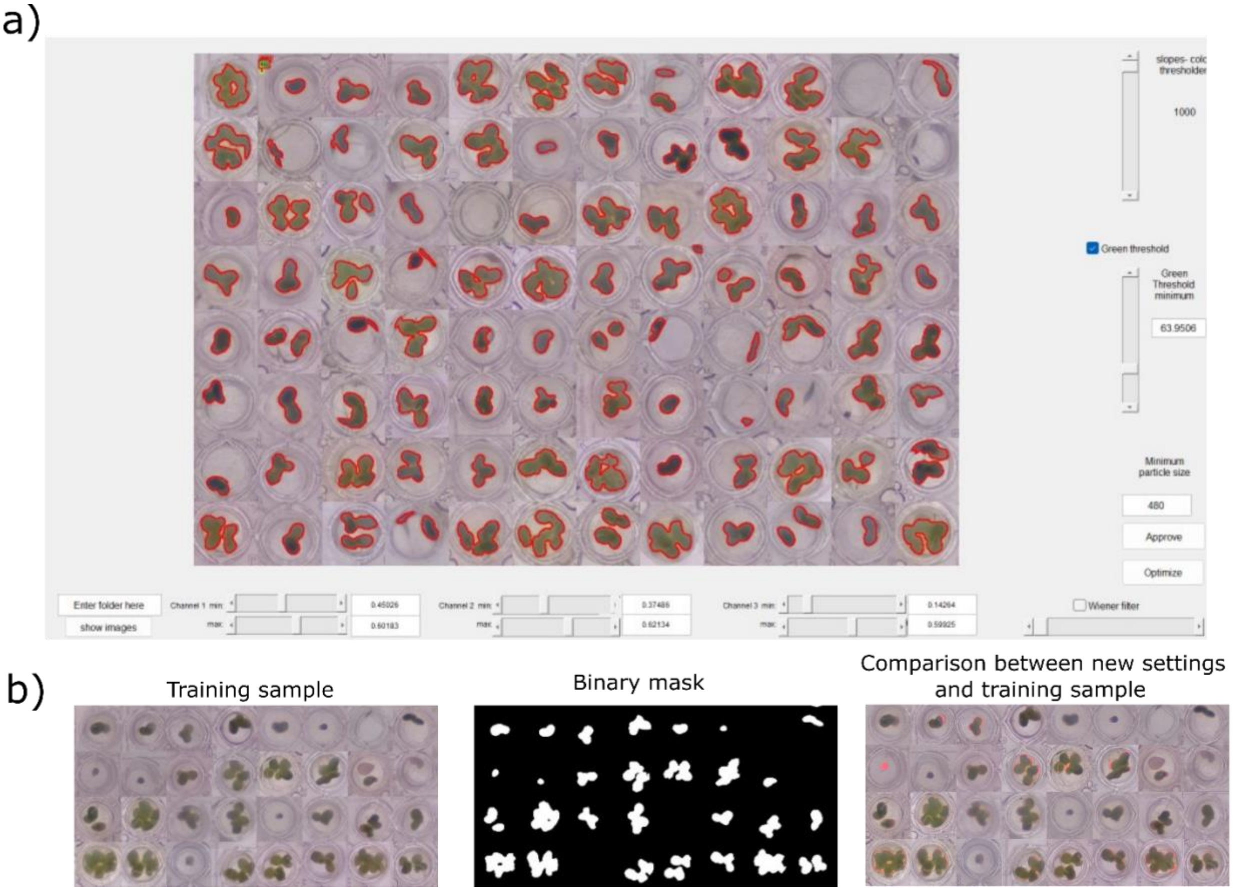

Fig. S7: *Optimization of duckweed detection. Here, we demonstrate our GUI to facilitate in the process of changing color threshold settings (a), followed by the training sample, binary mask for training sample, and a comparison between the performance of the optimal color threshold settings and the training sample, with areas of change in red (b).*

Experiment Design

Each experimental unit was a tiny microcosm in one well of a 96-well plate (Sarstedt, 83.3925), containing duckweeds, and one combined treatment of N, P, K, and microbe presence. Each well received 240 µL of liquid growth media and nutrients followed by either 5 µL of microbial inoculum (20,000 cells/µL) or 5 µL of sterile culture media. Microbe treatments were blocked by plate to prevent cross-contamination while nutrient combinations were randomized across wells.

Plates were sealed with two gas-permeable membranes (Sigma Aldrich, Breathe Easy, Z380059, and Sigma-Aldrich, Breathe Easier, Z763624) to prevent contamination and reduce the evaporation rate of well solutions. Plates were monitored for 10 days in an environmentally controlled growth chamber at 22°C and 150 μmol^2 lighting for 16 hours, and 18°C and dark for 8 hours. A total of 64 well plates were required to accommodate the high number of samples.

Preparation of Duckweed Samples

Duckweed samples were originally collected from Cedarvale Pond (43°41’23.0”N 79°25’10.0”W) in Toronto, Ontario, and were clonally propagated from a single frond unit. Microbes were collected from crushed plant tissue and incubated on yeast mannitol agar (YMA) plates at 30°C for two days followed by storage at 4°C. Following the isolation of microbes, plants were sterilized by treatment with 1% NaOCl for 60 seconds. The clonal, sterilized line was maintained in sterile growth media (recipe adapted from Krajnčič et al., 1995) in glass jars.

Models and Results Tables

To showcase the HTE system, we examined the effects of nitrogen levels on duckweed growth with and without microbes in our 6,000-unit experiment. We fit linear mixed models to the daily measurements of frond area and greenness, with the number of days since the start of the experiment, microbes (presence/absence), N level, and the interaction between microbes and N level as fixed effects. We fit N level and the number of days as linear and second order polynomial terms. Random effects included well plate and whether the well was located on the plate’s edge or interior of the plate.

Table S1: *Model results for frond area. Values are reported for fixed effects, with "×" indicating an interaction effect. The following were fit as linear and second order polynomial terms: N = $NaNO_3$ mg/L; Day = number of days since the start of the experiment.*

**Parameter**	**Estimate**	**Std. Error**	**Statistic**	***p***
Intercept	9.78	0.14	68.25	<0.001
Microbes	0.86	0.04	22.46	<0.001
N	26.54	6.78	3.92	<0.001
N^2	-25.63	6.78	-3.78	<0.001
Day	618.71	4.85	127.66	<0.001
Day^2	-222.52	4.85	-45.90	<0.001
Microbes × N	7.63	9.71	0.79	0.432
Microbes × N^2	23.24	9.70	2.40	0.017

Table S2: *Model results for the greenness of fronds. Values are reported for the fixed effects of each parameter, with "×" indicating an interaction effect between parameters. The following parameters were fitted as linear and second order polynomial terms: N = $NaNO_3$ mg/L; Day = number of days since the start of the experiment.*

**Parameter**	**Estimate**	**Std. Error**	**Statistic**	***p***
Intercept	0.33	1.80×10^{-3}	182.45	<0.001
Microbes	4.07×10^{-3}	8.34×10^{-5}	48.52	<0.001
N	-0.04	0.01	-2.80	0.005
N^2	-0.06	0.01	-3.99	<0.001
Day	1.51	0.01	143.05	<0.001
Day^2	-0.83	0.01	-78.45	<0.001
Microbes × N	0.09	0.02	4.47	<0.001
Microbes × N^2	0.05	0.02	2.51	0.012

Results for Greenness of Fronds

Frond greenness initially stalled, potentially due to the stress of moving from glass jars to well plates. Like frond area, frond greenness increased through time (positive effect of Day: $p < 0.001$, Table S2) but started to plateau around day 5 and decreased towards the end of the experiment (Figure S8, negative effect of Day^2: $p < 0.001$). The presence of microbes increased plant greenness ($p < 0.001$). Unlike frond area, higher levels of N led to lower plant greenness over time (N: $p = 0.005$ and N^2: $p < 0.1$). However, in inoculated plants, higher levels of N did not alter plant greenness, which also increased over time (Microbes × N: $p < 0.001$ and Microbes × N^2: $p = 0.012$). The addition of microbes removed the effects of increasing nitrogen levels (Figure S8).

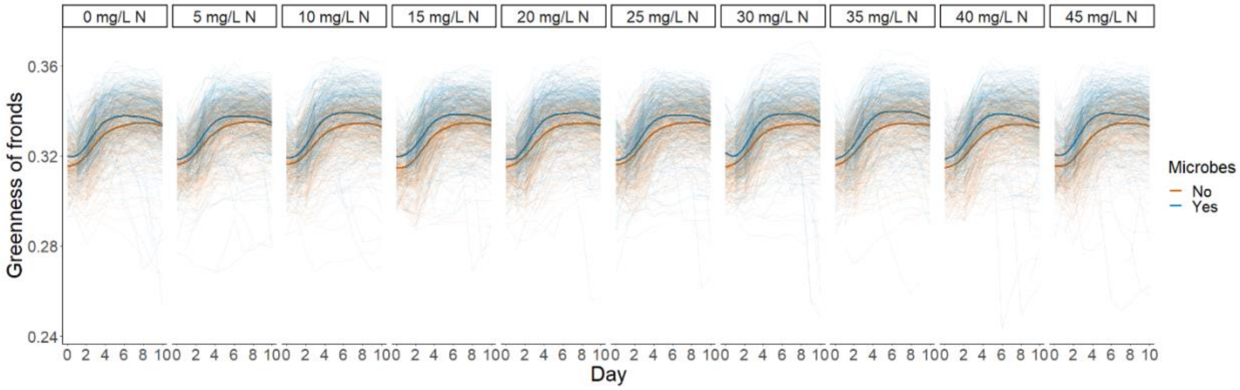

Figure S8: *Greenness of fronds, measured as the ratio of green values to the total RGB value for each pixel. Each line is frond area in one well. Darker lines depict the smoothed conditional means of plants inoculated with microbes (blue) and plants not inoculated with microbes (orange), averaged across other nutrient levels.*

References

Krajncic, B., Slekovec-Golob, M., & Nemec, J. (1995). Promotion of Flowering by Mn-EDDHA in the Photoperiodically Neutral Plant Spirodela polyrrhiza (L.) Schleiden. In *J. Plant Physiol* (Vol. 147).

Opentrons. (2022). *Opentrons Protocol Designer BETA*. https://designer.opentrons.com/
